# The Prognostic Significance of Tertiary Lymphoid Structures in Head and Neck Cancers: A Systematic Review and Meta‐Analysis

**DOI:** 10.1111/jop.70151

**Published:** 2026-05-13

**Authors:** Everton Freitas de Morais, Bruno Cesar da Costa, Antti Mäkitie, Ricardo D. Coletta, Alhadi Almangush

**Affiliations:** ^1^ Department of Oral Diagnosis, and Graduate Program in Oral Biology Piracicaba Dental School, University of Campinas São Paulo Brazil; ^2^ Laboratory of Biotechnology and Physiology of Reproduction Federal University of Ceara Sobral Ceará Brazil; ^3^ Department of Otorhinolaryngology, Head and Neck Surgery Helsinki University Hospital and University of Helsinki Helsinki Finland; ^4^ Research Program in Systems Oncology, Faculty of Medicine University of Helsinki Helsinki Finland; ^5^ Institute of Biomedicine, Pathology, University of Turku Turku Finland; ^6^ Department of Oral and Maxillofacial Diseases University of Helsinki Helsinki Finland; ^7^ Libyan Authority for Scientific Research Tripoli Libya

**Keywords:** head and neck squamous cell carcinoma, oral squamous cell carcinoma, prognostic biomarkers, survival analysis, tertiary lymphoid structures, tumor microenvironment

## Abstract

**Background:**

Head and neck squamous cell carcinoma (HNSCC), particularly oral squamous cell carcinoma (OSCC), is associated with poor survival despite therapeutic advances. Tertiary lymphoid structures (TLSs) are emerging components of the tumor microenvironment, but their prognostic significance in HNSCC remains unclear.

**Objective:**

To systematically review and meta‐analyze the prognostic impact of TLSs in HNSCC.

**Methods:**

PubMed/MEDLINE, Scopus, ScienceDirect, and Embase were searched through August 2025. Eligible studies evaluated TLS presence, density, maturity, or spatial distribution in histologically confirmed HNSCC. Pooled hazard ratios (HRs) with 95% confidence intervals (CIs) were calculated using fixed‐effects models. Risk of bias and certainty of evidence were assessed using QUIPS and GRADE, respectively.

**Results:**

Seventeen studies involving more than 2309 patients were included. TLS‐positive tumors were significantly associated with improved overall and disease‐free survival (DFS) (*p* < 0.001). Absence of TLSs was associated with worse overall survival (HR: 2.20, 95% CI: 1.74–2.78, *I* ^2^ = 26%) and poorer DFS (HR: 2.21, 95% CI: 1.49–3.28, *I* ^2^ = 0%). This adverse prognostic effect persisted in the OSCC subgroup (HR: 1.97, 95% CI: 1.42–2.73, *I* ^2^ = 28%). Beyond quantitative presence, TLS maturation and spatial distribution influenced prognosis, with mature intratumoral TLSs associated with more favorable outcomes. Moreover, emerging evidence suggests that TLSs may also predict response to immunotherapy, particularly immune checkpoint blockade.

**Conclusion:**

TLSs are strong prognostic biomarkers in HNSCC. Their presence, maturity, and spatial context significantly influence survival, supporting their role in prognostic stratification and future therapeutic strategies. TLS maturity emerges as a promising feature for standardized scoring systems and should be further explored in future studies.

## 1 Introduction

Head and neck squamous cell carcinoma (HNSCC) remains a major global health challenge, with approximately 389 000 new cases and 188 000 deaths annually [1]. Despite advances in surgical techniques, radiotherapy, as well as chemotherapy and immunotherapy, the overall five‐year survival rate remains unsatisfactory, at approximately 50%, and is significantly lower in advanced‐stage cases [1, 2]. This stagnant prognosis emphasizes the critical need for novel biomarkers capable of improving risk stratification, predicting therapeutic response, and guiding individualized management.

Within this context, the tumor microenvironment (TME) has emerged as a key determinant of oncological outcomes in HNSCC [3]. Immunological components of the TME, particularly tumor‐infiltrating lymphocytes and other stromal immune elements, have been extensively associated with prognosis and response to immunotherapy [4, 5]. Among these, tertiary lymphoid structures (TLSs), organized aggregates of lymphocytes that resemble secondary lymphoid organs, have gained increasing attention due to their capacity to coordinate local antitumor immunity [6, 7]. TLSs consist of B‐cell follicles, T‐cell zones, follicular dendritic cells, and high endothelial venules, supporting local antigen presentation and clonal expansion of effector immune cells [8, 9]. Their presence has been positively correlated with improved cytotoxic T‐cell infiltration, enhanced immune checkpoint blockade (ICB) therapy responsiveness, and superior overall survival (OS) across various malignancies, including HNSCC [10, 11, 12]. Recent studies have further implicated specific B‐cell subtypes, such as those expressing TCL1A, in the modulation and formation of TLSs and favorable OSCC prognosis [12]. However, the prognostic implications of TLSs in HNSCC remain inconsistently reported. Variability in TLS maturation stages, anatomical location (tumor core vs. invasive front), and detection methodology (e.g., hematoxylin–eosin staining vs. immunohistochemistry) contribute to conflicting results in the literature [13, 14]. For instance, while mature TLSs are generally associated with better outcomes, immature or disorganized TLSs may harbor immunosuppressive elements, such as regulatory T cells, that could contribute to immune evasion and poorer survival [11, 13]. Furthermore, existing histopathological staging systems for HNSCC fail to account for immune‐related variables such as TLSs, limiting their prognostic accuracy [10, 15, 16]. Given these complexities, there is an urgent need for a systematic synthesis of available data to clarify the prognostic relevance of TLSs and establish standardized assessment frameworks that could be integrated into routine clinical practice [17].

The objective of the present study was to systematically review and meta‐analyze the current evidence regarding the prognostic impact of TLSs in HNSCC. By consolidating and critically analyzing available evidence, this work contributes to elucidate the clinical significance of TLSs in HNSCC, highlighting their potential as biomarkers for prognostication and therapeutic guidance.

## 2 Materials and Methods

### 2.1 Study Design and Registration

This study followed the Preferred Reporting Items for Systematic reviews and Meta‐Analyses (PRISMA) guidelines [18] and was prospectively registered in the International Prospective Register of Systematic Reviews (PROSPERO) under the identification number CRD420251027267. The objective was to evaluate the prognostic value of TLSs in HNSCC, specifically regarding their association with survival outcomes such as OS, disease‐specific survival (DSS), and disease‐free survival (DFS). In line with the PICO framework, the population comprised patients with histopathologically confirmed HNSCC in which TLSs were assessed by histological and/or immunohistochemical methods; the intervention/exposure was defined as the presence, density, maturation, or spatial distribution of TLSs; the comparator consisted of patients with low TLS density or absence of TLSs; and the outcomes of interest included OS, DSS, and DFS.

### 2.2 Eligibility Criteria

Observational studies, such as prospective or retrospective cohort studies, case–control studies and clinical trials, that investigated the prognostic relevance of TLSs in histologically confirmed HNSCC were included. Eligible studies were required to assess the presence, density, maturity, or spatial distribution of TLSs using hematoxylin and eosin (H&E) staining, or immunohistochemistry (e.g., CD20, CD21, CD23, PNAd), and to report at least one extractable survival outcome. Studies were excluded if they provided only qualitative TLS assessment without quantitative classification, lacked a comparator group (TLS‐low/absent), or did not report survival data or hazard ratio (HR). Reviews, case reports, nonhuman studies, and conference abstracts without full data were also excluded.

### 2.3 Literature Search and Study Selection

A systematic search was conducted across four major electronic databases, including PubMed/MEDLINE, Scopus, ScienceDirect, and Embase, from inception until August 2025. The search strategy incorporated both controlled vocabulary and free‐text terms related to head and neck cancers, TLSs, and survival outcomes. No language or date restrictions were applied. The references of all included articles and relevant reviews were also screened manually for additional eligible studies. The detailed search keys are available in Supporting Information 1. Two independent reviewers conducted the title/abstract and full‐text screening, and disagreements during the selection process were resolved by consensus or through consultation with a third reviewer when necessary.

### 2.4 Data Extraction

Data were extracted independently by two reviewers using a standardized form that captured relevant study details, including author name, publication year, study design, country of origin, sample size, tumor subsite, HPV status when available, method of TLS detection, classification criteria (density, maturation, location), type of survival outcome reported, and the corresponding HR with 95% confidence interval (CI). All extracted data were cross‐validated by the reviewers to ensure accuracy.

### 2.5 Risk of Bias and Certainty of Evidence Assessment

The methodological quality and risk of bias of the included studies were assessed using the Quality in Prognosis Studies (QUIPS) tool, which is tailored to evaluate prognostic factor studies [19]. This tool considers six domains: study participation, study attrition, prognostic factor measurement, outcome measurement, study confounding, and statistical analysis and reporting. Each domain was evaluated independently by two reviewers and rated as low, moderate, or high risk of bias. Overall study quality was classified as low risk if all domains were scored as low or moderate, with at least four being low. Studies were considered high risk of bias if two or more domains received high‐risk ratings. Discrepancies were resolved through discussion.

The certainty of evidence for each prognostic marker was assessed using the Grading of Recommendations Assessment, Development and Evaluation (GRADE) methodology adapted for prognostic factor research [20].

### 2.6 Data Synthesis and Statistical Analysis

Meta‐analyses were conducted when at least three studies reported comparable effect measures for a given outcome. Pooled HRs and 95% CI were calculated using the inverse variance method under a fixed‐effects model, as implemented in Review Manager (RevMan) version 5.4.1. Adjusted HR from multivariate models was prioritized; however, if only unadjusted HRs were available, they were included with appropriate sensitivity analyses. Statistical heterogeneity was evaluated using the *I* ^2^ statistic, with values above 50% indicating moderate to substantial heterogeneity. Forest plots were generated to visually assess pooled effects and confidence intervals. The presence of TLS (TLS‐positive) was used as the reference category. To ensure consistency when studies reported results in opposite directions, the reciprocal transformation (1/HR) was applied to the inverted HR values.

## 3 Results

### 3.1 Characteristics of the Included Studies

A comprehensive literature search identified 561 records, with an additional 203 records retrieved through citation searching. After the removal of 82 duplicates, 479 records were screened, of which 445 were excluded because they were review studies, abstracts, editorial letters, or did not evaluate HNSCC or TLSs. A total of 39 full‐text reports were assessed for eligibility, and 22 were excluded for not evaluating survival outcomes and/or not directly assessing TLSs. The reasons for exclusion of these 22 full‐text articles are presented in Supporting Information 2.

**FIGURE 1:**
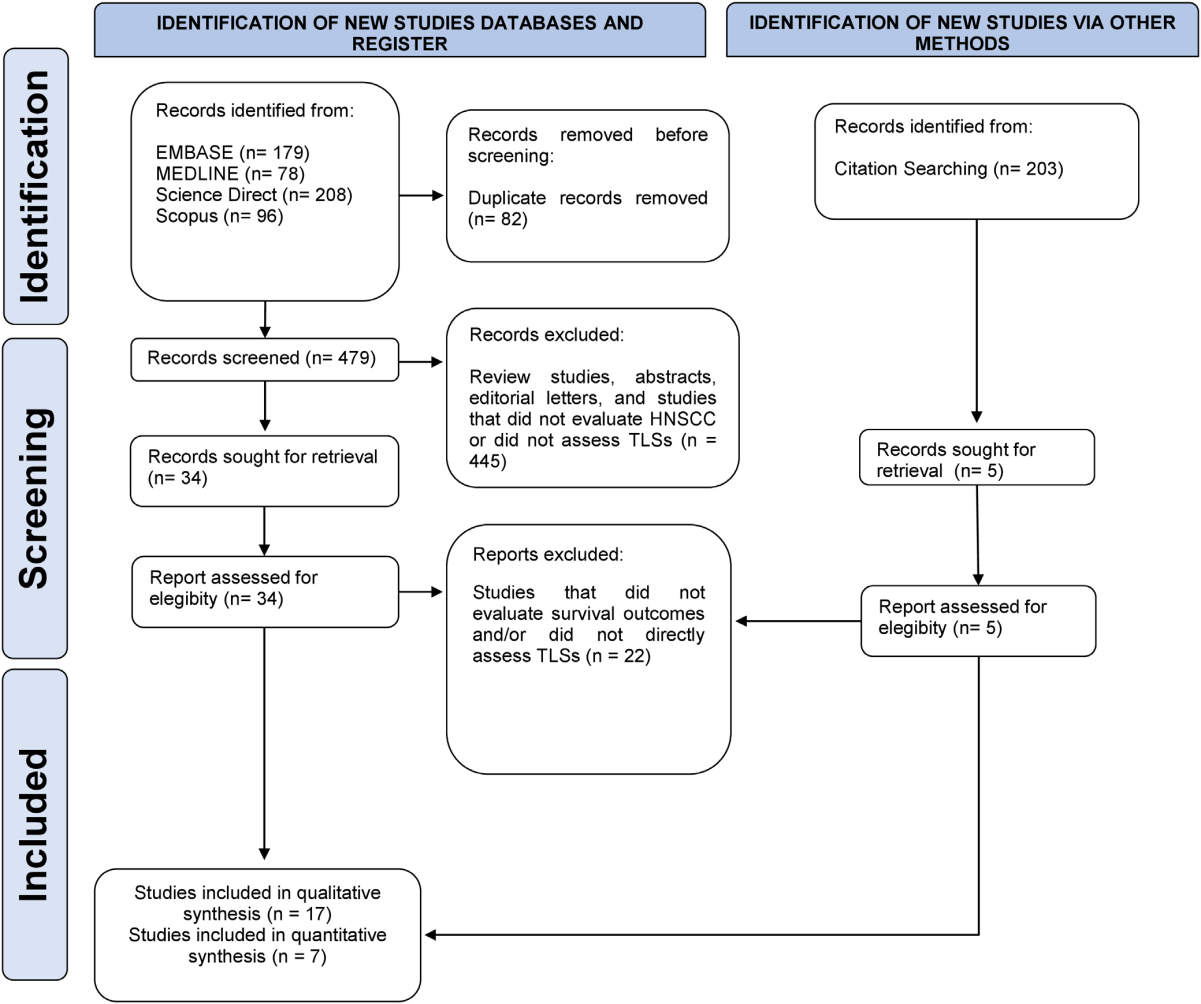
PRISMA flow diagram of literature search and selection criteria applied in this study.

Finally, 17 studies met the inclusion criteria and were incorporated into this systematic review [10, 15, 21, 22, 23, 24, 25, 26, 27, 28, 29, 30, 31, 32, 33, 34, 35] (Figure 1), encompassing a total of 2309 patients with HNSCC. The majority of the studies focused on OSCC or specifically on oral tongue squamous cell carcinoma (OTSCC), while others included squamous cell carcinomas arising from different regions of the head and neck. The evaluation of TLSs varied considerably across studies in terms of detection methodologies, operational definitions, and maturity classification. Most investigations relied on H&E staining for the morphological identification of TLSs, with several studies additionally employing immunohistochemical analyses using markers such as CD3, CD20, CD21, CD23, and BCL‐6 to confirm TLS presence and assess their maturation status. Of the 17 studies included in the systematic review, 7 provided sufficient data to be incorporated into the quantitative meta‐analysis (Table 1).

**TABLE 1:** Summary of studies included in the meta‐analysis reporting TLS in head and neck cancer.

Author (year)	Total number of participants	HNSCC subsites included	Tumor stage	TLS assessment method	TLS presence	Main results	Survival endpoint	Statistical values reported in the multivariate analysis (reference: TLS+)
Li et al. (2020) [23]	168	Tongue: 43.5% Buccal mucosa: 23.2% Gingiva: 22.0% Others: 11.3%	Not reported	IHC; TLS identified by PNAd, CD20, and CD3.	TLS+: 26.8%	TLS presence associated with markedly better overall and recurrence‐free survival.	OS DFS	3.78 [1.49, 9.59] 3.29 [1.28, 8.46]
Wang et al. (2021) [24]	97	Oral tongue squamous cell carcinoma	cT1N0: 36.1% cT2N0: 63.9% M stage: All patients M0 (early‐stage cohort)	H&E plus immunohistochemistry; TLS identified morphologically and characterized using CD3, CD20, CD21, Bcl‐6, and Bcl‐2.	TLS+: 76.3%	Higher TLS maturity strongly correlated with improved survival and lower recurrence.	OS DFS	2.30 [1.13, 4.68] 2.17 [1.11, 4.24]
Almangush et al. (2022) [10]	310	Oral tongue squamous cell carcinoma	T1N0M0: (30.7%); T2N0M0: 69.3%	H&E; TLS identified morphologically based on lymphoid aggregates and follicular structures.	TLS+: 84.8%	TLS‐positive tumors showed significantly better disease‐specific survival.	OS	1.66 [1.11, 2.48]
Wang et al. (2023) [27]	188	Tongue: 33.0% Gingiva15.4% Buccal mucosa: 28.2% Floor of mouth: 5.9% Others: 17.5%	I–II: 45.7% III–IV: 54.3%	H&E plus immunohistochemistry; TLS identified by CD20, CD3, and PNAd.	TLS+: 56.9%	TLS presence linked to substantially better overall and disease‐free survival.	OS DFS	4.03 [1.67, 9.73] 1.94 [1.10, 3.42]
Almangush et al. (2025) [15]	115	Nasopharyngeal carcinoma	I: 13% II: 25.2% III: 34.8% IV: 27%	H&E; TLS identified morphologically based on lymphoid aggregates and follicular structures.	TLS+: 55.7%	TLSs associated with improved overall and disease‐specific survival in nasopharyngeal carcinoma.	OS	1.68 [1.02, 2.77]
Li et al. (2025) [33]	327	Nasopharyngeal carcinoma (NPC)	III‐IV: 95.8%	H&E (morphological TLS definition) + IHC/mIHC for immune cell characterization + RNA‐based TLS gene signature.	TLS+: 45.8%	TLS positivity was an independent predictor of better survival, including under immunotherapy.	OS	3.84 [1.09, 13.53]
Xu et al. (2025) [35]	260	Tongue: 35.7% Buccal: 27.3% Gingiva: 12.7% Oropharynx: 21.2% Other: 3.1%	Stage I: 10.8% Stage II: 43.1% Stage III: 26.5% Stage IV: 19.6%	H&E (morphological TLS definition) + IHC/mIHC for immune cell characterization + RNA‐based TLS gene signature.	TLS+: 67.7%	Higher TLS immune score correlated with better survival across multiple HNSCC subsites.	OS	3.03 [1.72, 5.34]

Abbreviations: Bcl‐2, B‐cell lymphoma 2; Bcl‐6, B‐cell lymphoma 6; CD3, Cluster of differentiation 3; CD20, cluster of differentiation 20; CD21, cluster of differentiation 21; cT, clinical tumor stage; DFS, disease‐free survival; H&E, Hematoxylin and eosin; HNSCC, head and neck squamous cell carcinoma; IHC, immunohistochemistry; mIHC, multiplex immunohistochemistry; M0, no distant metastasis; N0, no regional lymph node metastasis; NPC, nasopharyngeal carcinoma; OS, overall survival; PNAd, peripheral node addressin; RNA, ribonucleic acid; TLS, tertiary lymphoid structures.

Across the 17 included studies, TLSs were identified mainly on H&E–stained sections, either alone or in combination with immunohistochemistry. Conventional H&E‐based histopathology was used in some early‐stage cohorts [10], whereas most oral cavity and laryngeal series supplemented H&E with single‐plex or multiplex IHC for canonical B‐, T‐cell and follicular dendritic‐cell markers such as CD3, CD8, CD20, CD21, CD23, PNAd, BCL‐6, and CD10 [21, 23, 24, 25, 26, 27, 32]. More advanced studies employed multiparametric imaging with CODEX [28], high‐plex multiplex immunofluorescence to map TLSs and surrounding immune cell populations [34], single‐cell RNA sequencing with mIHC validation to define TLS‐related chemokine‐receptor programs [29], and integration of bulk RNA‐seq–derived TLS gene signatures [22, 27, 33]. One study applied a convolutional neural network to automatically detect and quantify TLSs on whole‐slide H&E images from TCGA, with IHC validation of key TLS‐related genes [30], while another combined multiplex IHC with digital spatial transcriptomics using GeoMx DSP to characterize TLS maturity and localization in head and neck squamous cell carcinoma [35].

The maturity of TLSs was categorized in most studies using established frameworks that distinguish between early TLS (E‐TLS), primary follicular TLS (P‐TLS), secondary follicular TLS (S‐TLS), and TLS‐negative tumors. Follow‐up periods ranged from 24 to 120 months, and outcomes analyzed included OS, DFS, and associations with histopathological and immunological features.

### 3.2 Association Between TLS Presence and OS

**FIGURE 2:**
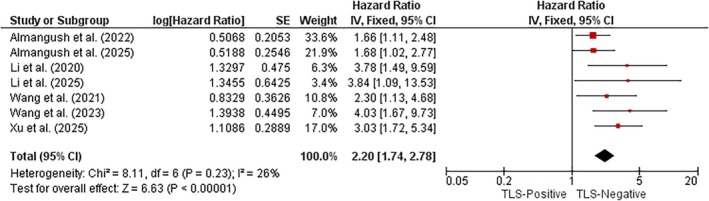
Forest plot of overall survival according to TLS status in HNSCC, with the TLS‐positive group serving as the reference category.

**FIGURE 3:**
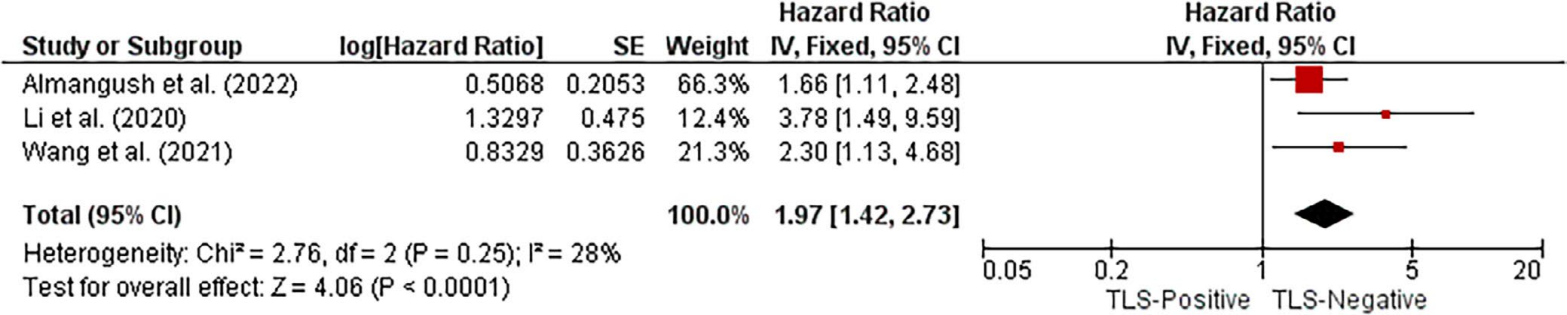
Forest plot of overall survival according to TLS status in OSCC. The TLS‐positive group was used as the reference category.

Seven studies provided data to the meta‐analysis of TLS presence and OS in HNSCC (Figure 2), while three studies provided data for the OSCC subgroup (Figure 3). The pooled analysis demonstrated that TLS‐negative tumors were significantly associated with worse OS, with a combined HR of 2.20 (95% CI: 1.74–2.78, *p =* 0.00001). Heterogeneity was low (*I* ^2^ = 26%), indicating consistency among the included studies. In subgroup analysis restricted to OSCC (three studies), the prognostic significance persisted, with a pooled HR of 1.97 (95% CI: 1.42–2.73, *p =* 0.0001) and similarly low heterogeneity (*I* ^2^ = 28%). Several studies further reported that the prognostic strength of TLSs was dependent on their maturation status and anatomical localization [24, 27, 32, 35]. For example, mature TLSs, particularly when located within or at the invasive margin of the tumor, were strongly predictive of better outcomes. In contrast, peritumoral TLSs or those classified as immature were sometimes associated with less favorable prognostic profiles, potentially reflecting differing immune functionalities [27].

One of the most comprehensive evaluations was conducted by Xu et al. [35], who applied spatial transcriptomic profiling to classify TLSs by location and maturity in a large OSCC cohort. Their findings demonstrated that intratumoral P‐TLS and S‐TLS were enriched in B‐cell receptor signaling, IL‐2, and NF‐κB pathways and associated with significantly improved OS. In contrast, mature TLSs located outside the tumor parenchyma displayed gene signatures associated with epithelial‐mesenchymal transition (EMT), angiogenesis, and TGF‐β signaling, correlating with worse survival. This spatial dichotomy highlighted the context‐dependent prognostic implications of TLSs. Similarly, Sun et al. [30] developed a convolutional neural network‐based algorithm to identify TLSs in histological slides, validating a gene expression signature composed of CXCR5, CD86, and CCR7 that was independently associated with OS and outperformed traditional staging models.

### 3.3 Association Between TLSs and DFS

**FIGURE 4:**
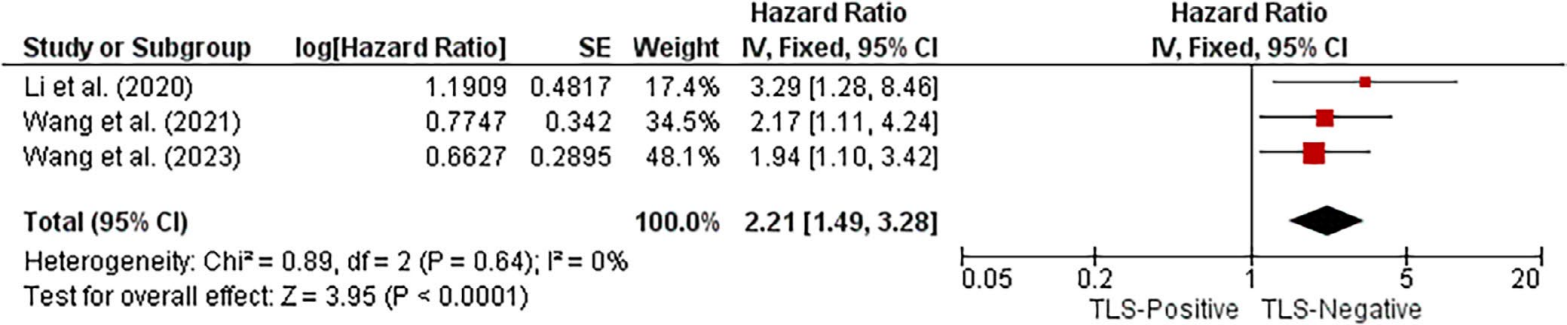
Forest plot of disease‐free survival according to intratumoral TLS status in HNSCC.

Three studies were eligible for meta‐analysis regarding DFS (Figure 4). TLS‐negative tumors were significantly associated with DFS with a pooled HR of 2.21 (95% CI: 1.49–3.28, *p* = 0.0001) and no observed heterogeneity (*I* ^2^ = 0%). The strongest evidence came from Wang et al. [24], who investigated 97 patients with early‐stage OTSCC. They reported that TLSs were independently associated with both improved OS and DFS in multivariable Cox regression models. Their data further showed that patients with immature TLSs and concurrent nodal metastasis had the worst prognosis, reinforcing the importance of TLS maturity in stratifying recurrence risk.

In addition to their prognostic value, TLSs were found to be associated with several clinicopathological variables. Higher TLS density and maturity were significantly associated with early T‐stage, absence of nodal metastases, lower histological grade, and reduced incidence of perineural and vascular invasion. These associations were consistent across multiple studies, including those by Weed et al. [28], Wu et al. [31], and Yao et al. [32], suggesting that TLSs may reflect a more organized and competent antitumor immune response that constrains local invasion and dissemination.

### 3.4 TLS Maturity, Spatial Distribution, and Immune Contexture

A recurrent theme across the studies was the critical role of TLS maturation and spatial location in determining their biological and prognostic impact. Mature TLSs, defined as those containing germinal centers and follicular dendritic cells, were found to be functionally active, with transcriptional evidence of B‐cell maturation, T follicular helper cell recruitment, and plasma cell differentiation. Xu et al. [35] showed that these structures, when located within the tumor mass, were associated with favorable immunological microenvironments, including increased infiltration of CD8+ T cells and M1 macrophages. Conversely, peritumoral TLSs with similar maturation features displayed signatures associated with tumor‐promoting processes such as immune exclusion and EMT.

### 3.5 Impact of TLSs on Therapeutic Response

Two studies evaluated the influence of TLSs on treatment outcomes. Seki et al. [26] examined patients with TSCC undergoing neoadjuvant chemotherapy with S‐1. While TLSs were not independently prognostic across the entire cohort, their presence significantly enhanced the therapeutic benefit of S‐1 when administered for more than 21 days, suggesting a potential synergy between TLS‐driven immunity and chemotherapeutic agents. Ruiz‐Torres et al. [34] analyzed a cohort of HNSCC patients receiving immune checkpoint inhibitors and reported that spatial proximity of TLSs to tumor cells was the strongest predictor of treatment response, as identified through machine‐learning algorithms incorporating over 500 immune, spatial, and molecular variables. In their analysis, TLS proximity outperformed established biomarkers such as PD‐L1 expression and tumor mutational burden, emphasizing the clinical utility of TLSs as predictive markers in the context of immunotherapy.

### 3.6 Risk of Bias and Quality Assessment of the Included Studies

The assessment of risk of bias and methodological quality of the included studies is presented in Supporting Information 3. Most studies were classified as having a moderate risk of bias, while a smaller proportion showed low risk, and only a minority presented serious methodological limitations. The domains most frequently affected were statistical analysis and reporting, and prognostic factor measurement, which frequently contributed to downgrading the overall QUIPS score. With respect to study participation and study attrition, the majority of studies were rated as moderate and low risk, respectively, suggesting that patient selection and follow‐up were generally adequate and well reported. In addition, many studies did not report whether evaluations were performed independently by more than one observer, which may compromise reproducibility and consistency of results. Another recurrent limitation was observed in the statistical reporting, since some studies failed to present HR and 95% CI, which reduces transparency and limits the precision of pooled analyses. When applying the GRADE framework, all studies were considered observational Phase 2 investigations, starting from a high level of certainty. After accounting for study limitations, imprecision, and indirectness, the certainty of evidence ranged between High and Moderate. Collectively, this indicates that, despite methodological shortcomings in specific domains, the overall body of evidence supporting the prognostic impact of TLS in HNSCC remains consistent and relatively robust.

## 4 Discussion

The results of the meta‐analysis demonstrated that the presence of TLSs is consistently associated with improved survival outcomes in HNSCC. Patients with TLS‐positive tumors showed significantly better OS and DFS, reinforcing the role of TLSs as strong prognostic biomarkers. These results align with accumulating evidence indicating that TLSs orchestrate adaptive antitumor responses and contribute to improved clinical outcomes across multiple malignancies [33, 36, 37]. Mature TLSs containing germinal centers and follicular dendritic cells actively support B‐cell maturation, plasma cell differentiation, and T follicular helper cell recruitment, thereby creating an immunologically “hot” microenvironment associated with favorable prognosis [36, 38].

The results of the study also highlight the relevance of TLSs in OSCC. Evidence from a multicenter study demonstrated that TLSs are prognostic markers in early OTSCC. Their presence was significantly associated with improved overall and DSS, independently of conventional prognostic factors such as TNM stage and WHO grading [10]. This reinforces the complexity of TLS assessment in OSCC and the need to integrate quantitative, qualitative, and spatial parameters into prognostic evaluations. The multicenter study by Almangush et al. [10] confirmed that TLSs provide prognostic value in early oral tongue carcinoma, independent of conventional clinicopathological parameters, further supporting their robustness across different stages of OSCC.

Recent molecular and spatial studies provide mechanistic insights supporting the findings of the current meta‐analysis. Single‐cell profiling has identified specific T‐cell subsets, such as PD‐1+CXCR5−CD4+ Th‐CXCL13 cells, which recruit B cells into TLSs and enhance antitumor immunity through IL‐21–mediated plasma cell differentiation [7]. Similarly, TCF1/TCF7+ T cells were shown to colocalize with TLSs in OSCC and predict improved survival, suggesting that TLSs act as niches sustaining stem‐like T cells with durable effector potential [8]. Moreover, B‐cell–centered signatures and germinal center formation within TLSs are increasingly recognized as major contributors to improved outcomes, particularly in HPV‐positive tumors [6]. These mechanistic links explain why TLS‐rich tumors not only display superior baseline survival but also respond better to ICB.

Another key aspect is the role of TLSs in immunotherapy response. The findings corroborate a previous study showing that intratumoral TLSs are predictors of improved outcomes after ICB in HNSCC [4]. TLS‐high tumors are characterized by enhanced TCR/BCR activation, antigen processing, and increased infiltration of CD8+ T cells, dendritic cells, and natural killer cells [4, 11]. Clinical and preclinical models have demonstrated that inducing TLSs, such as through LIGHT (TNFSF14) overexpression, can sensitize otherwise “cold” HPV‐negative HNSCC tumors to PD‐1 blockade [11]. Importantly, the spatial proximity of TLSs to tumor nests was identified as a decisive factor for therapy benefit [9, 34], and machine‐learning models suggest that TLS density may outperform PD‐L1 combined positive score as a predictor of ICB response [34]. However, there is a need for functional characterization of TLSs rather than merely quantifying their presence. In line with this, Mahindre et al. [14] suggested that TLSs may undergo dynamic changes during therapy, potentially shifting from immunostimulatory to immunosuppressive states depending on treatment context, which may influence resistance mechanisms. Taken together, these data emphasize the dual and context‐dependent nature of TLS biology. On one hand, our pooled results and several independent studies confirm that TLSs are associated with improved OS and DFS in HNSCC, supporting their use as reliable prognostic markers. On the other hand, heterogeneity in TLS maturation, anatomical distribution, and functional state raises caution in clinical translation, as immature or peritumoral TLSs may sustain tumor‐promoting processes.

Recent evidence from a large meta‐analysis of randomized clinical trials has shown that immune checkpoint inhibitors provide a modest but significant OS benefit in HNSCC, despite marked heterogeneity in clinical response and limited or inconsistent effects on progression‐free survival [39]. In this setting, TLS may represent a relevant biological determinant underlying such variability in immunotherapy outcomes. Accordingly, HNSCCs harboring TLS could be more likely to sustain effective immune activation following immunotherapy, whereas TLS‐poor tumors may contribute to primary resistance, partially explaining why only a fraction of patients derive durable benefit [40].

This evidence has important implications for clinical practice. Histological evaluation of TLSs could complement TNM staging and WHO grading in risk stratification of OSCC and HNSCC [10, 15]. Furthermore, TLS‐related gene signatures, identified by transcriptomic profiling, may serve as predictive biomarkers for immunotherapy and help refine patient selection [5, 17]. In the long term, therapeutic induction of TLSs may represent a novel immunomodulatory approach to enhance responses to ICIs, although strategies must consider the risk of generating dysfunctional or immunosuppressive TLSs. However, some limitations of our study should be acknowledged. The number of eligible studies for each endpoint was relatively small, particularly for DFS and OSCC subgroup analyses. Methodological heterogeneity in TLS evaluation across studies complicates data synthesis. Furthermore, the heterogeneity among HNSCC subsites may contribute to differences in TLS‐related outcomes, as tumors arising in distinct anatomical locations exhibit different etiological, microenvironmental, and immunological features that can directly influence TLS formation, maturation, and prognostic impact. Moreover, important clinicopathological factors such as HPV status were not consistently reported, despite strong evidence linking viral etiology to TLS abundance and functionality [6, 11]. Future research should therefore focus on standardizing TLS assessment, incorporating spatial and functional features, and prospectively validating TLSs as both prognostic and predictive biomarkers in HNSCC.

## 5 Conclusion

This systematic review and meta‐analysis demonstrated that the presence of TLSs is significantly associated with improved OS and DFS in HNSCC, including the OSCC subgroup. These findings propose TLSs as robust prognostic biomarkers and potential predictors of response to treatment. Although current evidence remains limited, TLS maturity has emerged as a promising parameter and warrants further investigation for potential incorporation into future standardized scoring systems. Standardization of assessment methods and prospective validation in clinical trials are needed to consolidate their role in patient stratification and as potential therapeutic targets.

## Author Contributions

**Everton Freitas de Morais and Bruno Cesar da Costa:** design, conduct, analysis, drafting of manuscript, presentation. **Antti Mäkitie:** design, conduct, analysis, and drafting of manuscript. **Ricardo D. Coletta:** design, conduct, analysis, and drafting of manuscript. **Alhadi Almangush:** design, conduct, analysis, drafting of manuscript.

## Funding

This research was funded by grants from Conselho Nacional de Desenvolvimento e Tecnológico (CNPq; grant 305781/2024‐3 to R. D. Coletta). E. F. de Morais (2022/00994‐5) and B. C. da Costa (2023/10632‐6) are research fellows supported by the Fundação de Amparo à Pesquisa do Estado de São Paulo‐FAPESP.

## Ethics Statement

The authors have nothing to report.

## Consent

The authors have nothing to report.

## Conflicts of Interest

The authors declare no conflicts of interest.

## Supporting information


**Supporting Information: 1.** Search strategy.

**Supporting Information: 2.** List of excluded studies along with reasons for exclusion (*n* = 22).

**Supporting Information: 3.** Risk of bias and methodological quality of the included studies.

**Data S1:** PRISMA 2020 checklist.

## Data Availability

The data that support the findings of this study are available from the corresponding author upon reasonable request.
