# Supplementary material for: The Prognostic Significance of Tertiary Lymphoid Structures in Head and Neck Cancers: A Systematic Review and Meta‐Analysis
Source: J Oral Pathol Med. 2026 May 13;55(7):774–83. doi: 10.1111/jop.70151 (PMC13429375; doi:10.1111/jop.70151)
Supplement: Supplementary file 1 — Supporting Information: 1. Search strategy. [file JOP-55-774-s001.docx]

**Supplementary Material 1.** Search strategy.

| Database | Search strategy |
| --- | --- |
| PubMed/Medline | #1: Head and Neck cancer*  ("Head and Neck Neoplasms"[Mesh] OR "Mouth Neoplasms"[Mesh] OR "Oropharyngeal Neoplasms"[Mesh] OR "Laryngeal Neoplasms"[Mesh] OR "Hypopharyngeal Neoplasms"[Mesh] OR ("head and neck cancer"[tiab] OR "HNSCC"[tiab] OR "oral squamous cell carcinoma"[tiab] OR "OSCC"[tiab] OR "oropharyngeal cancer"[tiab] OR "OPC"[tiab] OR "laryngeal cancer"[tiab] OR "hypopharyngeal cancer"[tiab] OR "nasopharyngeal cancer"[tiab] OR "NPC"[tiab]))    #2: Tertiary Lymphoid Structures*  "Tertiary Lymphoid Structures"[Mesh] OR “Tertiary Lymphoid Structure” OR “Tertiary Lymphoid Structures” OR “Tertiary Lymphoid Organ” OR “Tertiary Lymphoid Organs” OR “Ectopic Lymphoid Follicle” OR “Ectopic Lymphoid Follicles” OR “Ectopic Lymphoid Organ” OR “Ectopic Lymphoid Organs” OR “Intratumoral Lymphoid Structure” OR “Intratumoral Lymphoid Structures” OR “Intratumoral Lymphoid Organ” OR “Intratumoral Lymphoid Organs” OR “Intratumoral Lymphoid Follicles” OR “Intratumoral Lymphoid Follicle” OR “Intra-tumoral Lymphoid Structure” OR “Intra-tumoral Lymphoid Structures” OR “Intra-tumoral Lymphoid Organ” OR “Intra-tumoral Lymphoid Organs” OR “Intra-tumoral Lymphoid Follicles” OR “Intra-tumoral Lymphoid Follicle” OR “tumor-associated immune aggregates” OR “tumor associated immune aggregates”    #3: Prognosis*  ("Survival"[Mesh] OR "Disease-Free Survival"[Mesh] OR "Survival Analysis"[Mesh] OR "Survival Rate"[Mesh] OR "Progression-Free Survival"[Mesh] OR “Survival” OR “Survival Analysis” OR “Survival Rate” OR “Overall Survival” OR “OS” OR “Disease free survival” OR "Disease-Free Survival" OR “DFS” OR “Progression free survival” OR "Progression-Free Survival" OR “PFS” OR "Prognosis"[Mesh] OR Prognosis OR Prognostic) |
| Embase | #1: Head and Neck cancer*  ('head and neck tumor'/exp OR 'mouth tumor'/exp OR 'oropharynx tumor'/exp OR 'larynx tumor'/exp OR ('head and neck cancer':ti,ab OR 'HNSCC':ti,ab OR 'oral squamous cell carcinoma':ti,ab OR 'OSCC':ti,ab OR 'oropharyngeal cancer':ti,ab OR 'laryngeal cancer' OR 'hypopharyngeal cancer' OR 'nasopharyngeal cancer':ti,ab))    #2: Tertiary Lymphoid Structures  “Tertiary Lymphoid Structure” OR “Tertiary Lymphoid Structures” OR “Tertiary Lymphoid Organ” OR “Tertiary Lymphoid Organs” OR “Ectopic Lymphoid Follicle” OR “Ectopic Lymphoid Follicles” OR “Ectopic Lymphoid Organ” OR “Ectopic Lymphoid Organs” OR “Intratumoral Lymphoid Structure” OR “Intratumoral Lymphoid Structures” OR “Intratumoral Lymphoid Organ” OR “Intratumoral Lymphoid Organs” OR “Intratumoral Lymphoid Follicles” OR “Intratumoral Lymphoid Follicle” OR “tumor-associated immune aggregates” OR “tumor associated immune aggregates”    #3: Prognosis    ('survival'/exp OR 'disease free survival'/exp OR 'progression free survival'/exp OR 'prognosis'/exp OR 'survival':ti,ab OR 'survival analysis':ti,ab OR 'survival rate':ti,ab OR 'overall survival':ti,ab OR 'os':ti,ab OR 'disease free survival':ti,ab OR 'dfs':ti,ab OR 'progression free survival':ti,ab OR 'pfs':ti,ab OR 'prognosis':ti,ab OR 'prognostic':ti,ab) |
| Scopus | TITLE-ABS-KEY  #1: Head and Neck cancer  ("head and neck cancer" OR "HNSCC" OR "oral squamous cell carcinoma" OR "OSCC" OR "oropharyngeal cancer" OR "laryngeal cancer" OR "hypopharyngeal cancer" OR "nasopharyngeal cancer")    #2: Tertiary Lymphoid Structures  (Tertiary Lymphoid Structure OR Tertiary Lymphoid Structures OR Tertiary Lymphoid Organ OR Tertiary Lymphoid Organs OR Ectopic Lymphoid Follicle OR Ectopic Lymphoid Follicles OR Ectopic Lymphoid Organ OR Ectopic Lymphoid Organs OR Intratumoral Lymphoid Structure OR Intratumoral Lymphoid Structures OR Intratumoral Lymphoid Organ OR Intratumoral Lymphoid Organs OR Intratumoral Lymphoid Follicles OR Intratumoral Lymphoid Follicle OR Intra-tumoral Lymphoid Structure OR Intra-tumoral Lymphoid Structures OR Intra-tumoral Lymphoid Organ OR Intra-tumoral Lymphoid Organs OR Intra-tumoral Lymphoid Follicles OR Intra-tumoral Lymphoid Follicle OR tumor-associated immune aggregates OR tumor associated immune aggregates)    #3: Prognosis    ("Survival" OR "Disease-Free Survival" OR "Survival Analysis" OR "Survival Rate" OR "Progression-Free Survival" OR “Survival” OR “Survival Analysis” OR “Survival Rate” OR “Overall Survival” OR “OS” OR “Disease free survival” OR "Disease-Free Survival" OR “DFS” OR “Progression free survival” OR "Progression-Free Survival" OR “PFS” OR "Prognosis"[Mesh] OR Prognosis OR Prognostic) |
| Science Direct | #1: Head and Neck cancer  ("head and neck cancer" OR "HNSCC" OR "oral squamous cell carcinoma" OR "OSCC" OR "oropharyngeal cancer" OR "laryngeal cancer" OR "hypopharyngeal cancer" OR "nasopharyngeal cancer")      #2: Tertiary Lymphoid Structures  (("Tertiary Lymphoid Structure" OR "Tertiary Lymphoid Structures" OR "Tertiary Lymphoid Organ" OR "Tertiary Lymphoid Organs" OR "Ectopic Lymphoid Follicle" OR "Ectopic Lymphoid Follicles" OR "Ectopic Lymphoid Organ" OR "Ectopic Lymphoid Organs" OR "Intratumoral Lymphoid Structure" OR "Intratumoral Lymphoid Structures" OR "Intratumoral Lymphoid Organ" OR "Intratumoral Lymphoid Organs" OR "Intratumoral Lymphoid Follicle" OR "Intratumoral Lymphoid Follicles" OR "tumor-associated immune aggregates" OR "tumor associated immune aggregates")    #3: Prognosis    ("Survival" OR "Disease-Free Survival" OR "Survival Analysis" OR "Survival Rate" OR "Progression-Free Survival" OR "Overall Survival" OR "OS" OR "Disease free survival" OR "DFS" OR "Progression free survival" OR "PFS" OR "Prognosis" OR "Prognostic" ) |
