# Supplementary material for: The Prognostic Significance of Tertiary Lymphoid Structures in Head and Neck Cancers: A Systematic Review and Meta‐Analysis
Source: J Oral Pathol Med. 2026 May 13;55(7):774–83. doi: 10.1111/jop.70151 (PMC13429375; doi:10.1111/jop.70151)
Supplement: Supplementary file 2 — Supporting Information: 2. List of excluded studies along with reasons for exclusion (n = 22). [file JOP-55-774-s004.docx]

Supplementary Material 2. List of excluded studies along with reasons for exclusion (n = 22).

| **Author (Year)** | **Title and DOI/PMID** | **Reason for Exclusion** |
| --- | --- | --- |
| Fridman et al. (2011) | Immune infiltration in human cancer: prognostic significance and disease control.  DOI: 10.1007/82_2010_46 | Review. |
| Economopoulou et al. (2021) | B cells and their role in shaping the immune response in squamous cell carcinoma of the head and neck.  DOI: 10.2217/imt-2021-0070 | Editorial. |
| Li et al. (2021) | PD-1⁺CXCR5⁻CD4⁺ Th-CXCL13 cell subset drives B cells into tertiary lymphoid structures of nasopharyngeal carcinoma.  DOI:10.1136/jitc-2020-002101 | Did not evaluate survival outcomes. |
| Peng et al. (2021) | Single-cell profiling of tumor-infiltrating TCF1/TCF7+ T cells reveals a T lymphocyte subset associated with tertiary lymphoid structures and superior prognosis in oral cancer.  DOI: 10.1016/j.oraloncology.2021.105348 | TLSs were not assessed as an independent prognostic variable, and the survival analysis focused on TCF7+ T-cell frequency rather than TLS presence, density, or maturity. |
| Ruffin et al. (2021) | B cell signatures and tertiary lymphoid structures contribute to outcome in head and neck squamous cell carcinoma.  DOI: 10.1038/s41467-021-23355-x | TLSs were not evaluated histologically nor analyzed as independent prognostic variables; survival outcomes were based on B-cell transcriptional signatures rather than TLS presence, density, or maturity. |
| Krupar (2022) | The tumor microenvironment-relay station for prognosis and therapy response.  DOI: 10.1007/s00292-022-01159-0 | Review. |
| Bao et al. (2023) | Exploring the dual role of B cells in solid tumors: implications for head and neck squamous cell carcinoma.  DOI: 10.3389/fimmu.2023.1233085 | Review. |
| Chu et al. (2023) | Pan-cancer T Cell Atlas Links a Cellular Stress Response State to Immunotherapy Resistance.  DOI: 10.1038/s41591-023-02371-y | This study does not investigate TLSs in head and neck cancer. |
| Li et al. (2023) | Tertiary Lymphoid Structure Raises Survival and Immunotherapy in HPV–HNSCC.  DOI: 10.1177/00220345231151685 | TLS analyses were primarily based on TCGA transcriptomic signatures, mechanistic mouse models, and in vitro/flow cytometry assays rather than direct detection and prognostic evaluation of TLSs in human tumor sections. |
| Liu et al. (2023) | Intratumoral tertiary lymphoid structures promote patient survival and immunotherapy response in head and neck squamous cell carcinoma.  DOI: 10.1007/s00262-022-03310-5 | TLSs were not assessed by histopathology or IHC in human tumor sections; prognostic analyses were derived from transcriptomic TLS signatures using TCGA datasets, without direct morphological identification or maturity classification of TLSs. |
| Ning et al. (2023) | ABCB11 accumulated in immature tertiary lymphoid structures predicts resistance to PD-1/PD-L1 inhibitors in HNSCC.  DOI: 10.1016/j.tranon.2023.101747 | TLSs were analyzed only to study molecular mechanisms of immunotherapy resistance; although HE/mIF identified TLS maturity, the study did not evaluate survival outcomes based on TLS morphology nor assess the prognostic impact of TLSs themselves. |
| Cha et al. (2024) | Single-cell analysis reveals cellular and molecular factors counteracting HPV-positive oropharyngeal cancer immunotherapy outcomes.  DOI: 10.1136/jitc-2023-008667 | This study does not investigate TLSs in head and neck cancer. |
| Lee et al. (2024) | Deciphering head and neck cancer microenvironment: Single-cell and spatial transcriptomics reveals HPV-associated differences.  DOI: 10.1002/jmv.29386 | TLSs were identified descriptively using spatial transcriptomics and limited IHC validation, but no survival analysis was performed based on TLS. |
| Mahindre et al. (2024) | Tertiary lymphoid structure in oral squamous cell carcinoma: An institutional experience.  DOI:10.1016/j.oor.2024.100281 | Did not evaluate survival outcomes; TLSs were identified only descriptively and without prognostic analysis (no OS, DFS, RFS, DSS, or hazard ratios). |
| Patysheva et al. (2024) | Revealing molecular mechanisms of early-onset tongue cancer by spatial transcriptomics.  DOI: 10.1038/s41598-024-76044-2 | Excluded because the study does not evaluate TLSs in relation to survival outcomes. |
| Sadeghirad et al. (2024) | Spatial dynamics of tertiary lymphoid aggregates in head and neck cancer: insights into immunotherapy response.  DOI: 10.1186/s12967-024-05409-y | This study investigated TLSs exclusively in recurrent/metastatic HNSCC and focused on predicting immunotherapy response, without analyzing survival outcomes (OS, DFS, DSS) in relation to TLS presence, density, or maturity. Therefore, it does not meet the eligibility criteria requiring prognostic survival analysis linked to TLSs. |
| Sosa et al. (2024) | Architecture of head and neck squamous cell carcinoma tumor microenvironment revealed: can tertiary lymphoid structures predict post-surgery recurrence?  DOI: 10.21037/tcr-23-2098 | This article is an editorial commentary, not an original research study. |
| Xie et al. (2024) | TCL1A-expressing B cells are critical for tertiary lymphoid structure formation and the prognosis of oral squamous cell carcinoma.  DOI: 10.1186/s12967-024-05292-7 | The study does not evaluate the prognostic impact of TLSs themselves. TLSs appear only as part of exploratory mechanistic analyses of B-cell function, without quantitative assessment of TLS density, maturity, or distribution, and without survival analyses stratified by TLS status. |
| Xing et al. (2024) | Tertiary Lymphoid Structures Gene Signature Predicts Prognosis and Immune Infiltration Analysis in Head and Neck Squamous Cell Carcinoma.  DOI: 10.2174/13892029278082240118053857 | This study evaluated a TLS-related gene signature using TCGA and GEO datasets but did not directly assess TLSs in tumor tissues (no histological or immunohistochemical identification). |
| Zhu et al. (2024) | Tertiary lymphoid structures in head and neck squamous cell carcinoma.  DOI: 10.1016/j.tranon.2024.101949 | Review. |
| Zhang et al. (2025) | A novel tertiary lymphoid structure-associated signature accurately predicts patient prognosis and facilitates the selection of personalized treatment strategies for HNSCC.  DOI: 10.3389/fimmu.2025.1551844 | This study evaluates a TLS-associated gene signature using TCGA and GEO datasets but does not directly assess TLSs in tumor tissue. |
| Lai et al. (2026) | The multidimensional regulatory network of the PD‑1/PD‑L1 axis in the esophageal squamous cell carcinoma microenvironment: Implications for novel combination therapies and precision immunotherapy (Review).  DOI: 10.3892/or.2025.9021 | Review. |
